# A Systematic Evaluation of Measures Against Highly Pathogenic Avian Influenza (HPAI) in Indonesia

**DOI:** 10.3389/fvets.2019.00033

**Published:** 2019-02-18

**Authors:** Muchammad Gumilang Pramuwidyatama, Henk Hogeveen, Helmut W. Saatkamp

**Affiliations:** ^1^Business Economics Group, Wageningen University and Research, Wageningen, Netherlands; ^2^Department of Farm Animal Health, Faculty of Veterinary Medicine, Utrecht University, Utrecht, Netherlands

**Keywords:** HPAI H5N1, endemic, evaluation, measures, mitigation, strategy, vaccination

## Abstract

Over the past years, many different control measures have been implemented to prevent HPAI infection. The national plan with numerous measures lead to problems in terms of prioritization and budget allocation. Our study objectives are to (i) establish an inventory of measures on HPAI control in Indonesia since the first actions were taken in 2004, (ii) evaluate preferences for different HPAI control measures applied in the West Java province at the district level during 2013–2017, and (iii) establish a basis for further qualitative and quantitative research to improve control for an endemic HPAI in Indonesia. This research was carried out according to the following five steps (i) development of an HPAI management framework for an endemic state, (ii) inventorization of measures directed at HPAI and description of the development of HPAI in Indonesia, (iii) development of a questionnaire for the experts involved, (iv) systematic evaluation of preferences for short- and long-term HPAI strategies and measures applied in the West Java Province based on expert opinion, and (v) data analysis. The study systematically evaluated in total 27 measures. The results of this study show that the animal disease management framework is helpful as a systematic structure to distinguish and evaluate strategies and measures. In our framework, we defined the following strategies: prevention, monitoring, control, mitigation, eradication, and human protection. The findings of our research show that the primary aims of the government were to safeguard humans from HPAI transmission by mitigating HPAI disease in livestock. The measures with the highest priority were preventive vaccination of poultry, biosecurity, and stamping-out infected flocks. This showed that the government predominantly chose a vaccination-based HPAI mitigation strategy. However, the chosen strategy has a low implementation feasibility. A collaboration between the responsible stakeholders farmers may increase the feasibility of the chosen strategy in the future. Furthermore, our findings provide a basis for research into the motivation of farmers to implement different measures as well as into the expected impact of different measures to develop an effective and efficient mitigation approach.

## Introduction

The first major outbreak of Highly Pathogenic Avian Influenza (HPAI) H5N1 in Indonesia was in December 2003 (1). Since then, HPAI remained endemic in most regions in the country. HPAI is a zoonotic disease that severely infects both poultry and humans and has a high mortality rate [(2), p. 243]. During the outbreaks in 2003–2004, the Indonesian poultry industry suffered a loss of millions of US dollars through the death of millions of chickens and costs to control the spread of the disease [(3), p. 8]. Many small-scale poultry farmers stopped their activities and, as a consequence, lost their primary source of income, because the risk of infection was too high. Furthermore, there were 200 human-HPAI cases of which 168 were lethal (4).

The Indonesian government decided to regard HPAI H5N1 as one of the top priority zoonotic diseases due to the magnitude of its potential impact on the poultry industry and public health. A national strategic plan with measures to mitigate the HPAI epidemic was launched in 2006 [(5), p. 39–51). The formulation and implementation of the plan involved parties from ministries and international agencies, such as the Food and Agriculture Organization, World Organization of Animal Health (OIE), and the World Health Organization.

Over the past years, many different control measures have been implemented to prevent HPAI infection. The national plan with numerous measures lead to problems in terms of prioritization and budget allocation. In Indonesia, the autonomous district governments are mainly responsible for controlling HPAI, based on national guidelines. This decentralization has been argued to be an important challenge of controlling HPAI in Indonesia, because district governments may have their own judgement of measures to be implemented based on available financial and human resources as well as local support (6). Consequently, it is difficult to systematically evaluate the efficacy of each measure. In addition, academic literature mostly focuses on specific technical measures to control the disease either directly or indirectly, for instance, vaccination (7, 8), or participatory disease surveillance, and response (9). Considering the complexity of the issue, involving not only the HPAI virus but also animals and human actors (e.g., farmers, government), it is necessary to have a systematic evaluation of measures aimed to control HPAI. Currently, such an all-encompassing, systematic evaluation is lacking in Indonesia.

This paper aims to fill this gap by providing a systematic evaluation of HPAI control strategies in Indonesia. The objectives of this study are to (i) establish an inventory of measures on HPAI control in Indonesia since the first actions were taken in 2004, (ii) evaluate preferences for different HPAI control measures applied in the West Java province at the district level during 2013–2017, and (iii) establish a basis for further qualitative and quantitative research to improve HPAI control strategies in an endemic state in Indonesia.

## Materials and Methods

**Figure 1:**
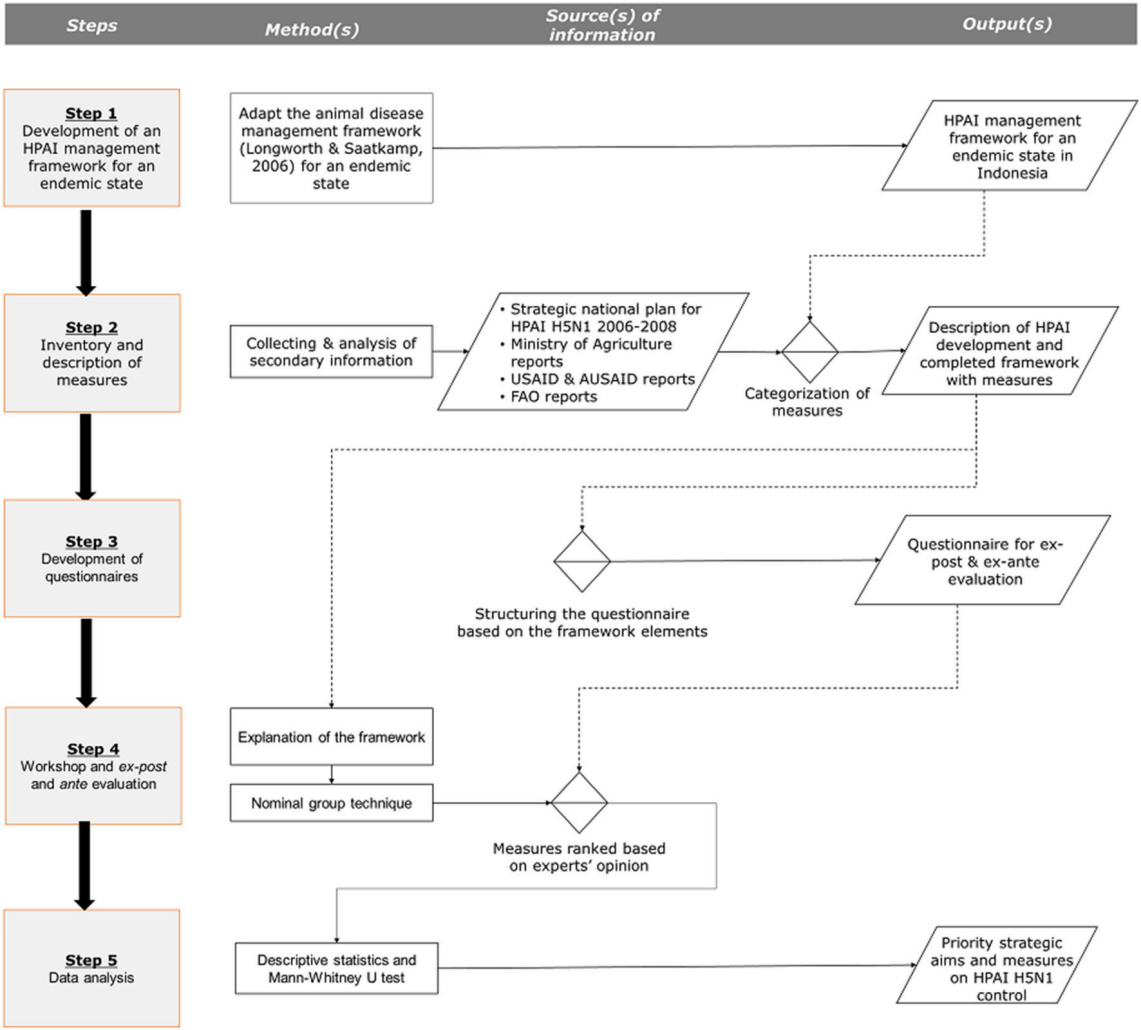
Steps and approaches for the evaluation of measures directed at HPAI mitigation in Indonesia.

This research was carried out according to the following five steps (Figure 1): (i) development of an HPAI management framework for an endemic state, (ii) inventorization of measures directed at HPAI and description of the development of HPAI in Indonesia, (iii) development of a questionnaire for the experts involved, (iv) systematic evaluation of preferences for short- and long-term HPAI strategies and measures applied in the West Java Province, based on expert opinion, and (v) data analysis.

### Step 1: Development of the HPAI Management Framework

Management of HPAI, particularly in an endemic state, is complex. This complexity makes it difficult to evaluate the efficacy of different measures, both for livestock and for the human health sector. A framework of animal disease management in the context of HPAI is essential to overcome this issue, especially in countries with endemic infections, such as Indonesia. In this study, we adapted the animal disease management framework of (10) to (i) provide a systematic inventory procedure for measures on HPAI in Indonesia, (ii) develop a questionnaire for evaluation, and (iii) combine the two into a systematic framework for measure evaluation.

**Figure 2:**
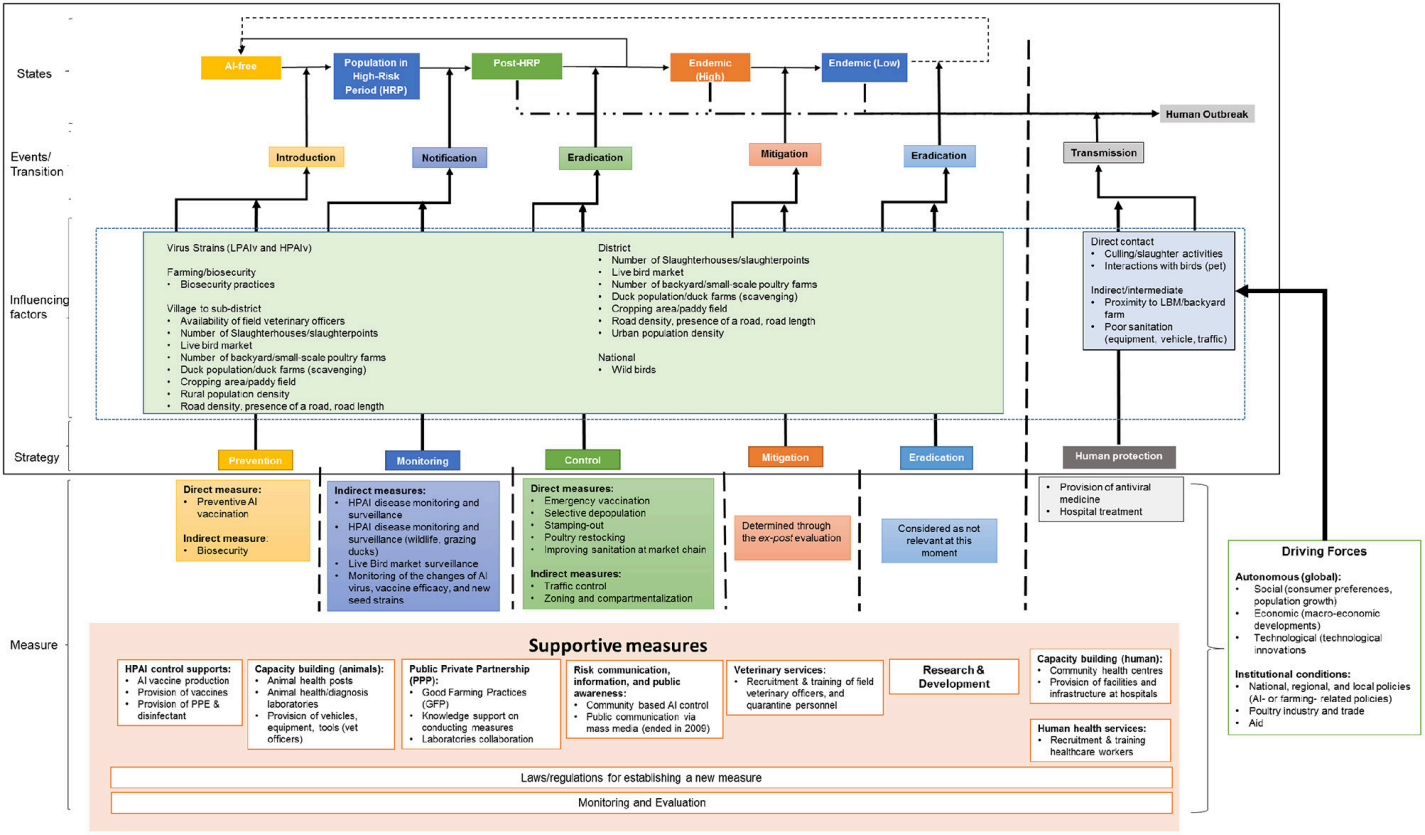
HPAI management framework for the endemic state in Indonesia.

Figure 2 depicts the HPAI management framework for an endemic HPAI that consists of six elements: *states, events* (i.e., the transition between states)*, influencing factors* (i.e., factors that determine the probability and time interval with which a population remains in a given state or enters into a different state), *driving forces* (i.e., factors that both directly and indirectly influence the implementation of measures), *strategy* (i.e., a group of measures aimed at a particular event with the same purpose), and *measures* (i.e., an activity with one or more specific aims).

In comparison with the original framework, two additional *states* were added. The adapted framework consists of five mutually exclusive states: (i) AI-free, (ii) high-risk period (HRP), (iii) outbreaks (post-HRP), (iv) endemic (high), and (v) endemic (low). Since the nature of HPAI is zoonotic, we added an additional exhaustive state: (vi) human outbreak.

The AI-free state is defined as a district free of AI disease. The high-risk period (HRP) is defined as the period when the AI virus is present and can spread freely, but is not yet identified in a given district. Once HPAI has been identified—either through monitoring and surveillance activities or by a clinical outbreak in livestock or humans—the state of the outbreak (post-HRP) starts. The endemic state is defined as a state when there is a constant presence of AI cases or outbreaks within part of a district. In the context of this study, the endemic state is subdivided into two states: high or low HPAI prevalence. More specifically, as there is a lack of accurate data or reports about HPAI outbreaks, we defined a district with high HPAI prevalence as one where outbreaks occur in more than 50% of the sub-districts or when HPAI is identified in AI-free sub-districts. A low-prevalence endemic state is defined as a state where outbreaks of HPAI occur in < 50% of the sub-districts, while AI-free sub-districts remain free of HPAI. The human outbreak state is defined as one or more human HPAI infections in a given district.

The framework includes five *events*: introduction, notification, eradication, mitigation, and transmission. Introduction means that the HPAI virus entered into an AI-free district. The presence of HPAI virus is detected and notified within the event of notification. After the disease has been notified, eradication will follow in which ideally control measures are applied to reduce the prevalence of HPAI to zero. When actions to eradicate are not sufficient, mitigation measures are put in place to reduce the prevalence of HPAI until control measures can eradicate the virus effectively. The event of transmission represents the passing of HPAI from animals to humans.

*Influencing factors* can be further divided into factors that are responsible for HPAI transmission either in livestock or in humans. A number of influencing factors of HPAI transmission in livestock have previously been identified, such as: rice cropping intensity, precipitation, farming/trade intensity, low elevation, road density, and backyard farm population [(11), p. 4–5; (12), p. 2–5; (13), p. 4–7; (14), p. 3–7). Likewise, several risk factors of HPAI transmission to humans have been identified, such as direct and indirect contact with a sick or dead bird, visiting a wet or live bird market, consuming sick poultry, and poor sanitation [(15), p. 1843–1845; (16), p. 1728–1733].

*Driving forces* can be either autonomous (global) or institutional. Autonomous driving forces include macroeconomic developments that have no direct link with the poultry industry, while institutional driving forces are local to national policies, which in this case are related to the Indonesian poultry industry and HPAI itself.

Coping with a disease event requires a *strategy*. Three additional strategies directed to HPAI were added to the original framework of (10) consists prevention, monitoring, and control in the EU context. While our adapted framework recognizes the endemic state and human-HPAI cases in Indonesia, therefore strategies of mitigation (i.e., minimize the number of outbreaks), eradication [(17), p. 1], and human protection [(5), p. 23–30] were added to the adapted framework. The strategy of prevention is a combination of measures that aim to reduce the likelihood of disease introduction into a domestic population. The strategy of monitoring is a combination of measures aimed to monitor and surveil a population to reduce the high-risk period. The strategy of control is a combination of measures aimed to eradicate the disease as quickly as possible. The strategy of mitigation involves a combination of measures aimed to reduce the prevalence of a disease to the extent that control measures can effectively eradicate the disease. The strategy of eradication involves a combination of measures aimed to completely eradicate a disease that already has a low prevalence. Both mitigation and eradication strategies consist of a combination of measures from prevention, monitoring, and control strategies. In our framework (Figure 2), mitigation and eradication strategies are intentionally left blank because district governments have the autonomy to implement different combinations of measures to suit the local context. The human protection strategy comprises a combination of measures aimed at reducing the risk of HPAI transmission from animals to humans and at treating HPAI patients. Within the context of this study, we focused on mitigation, eradication, and human protection strategies.

*Measures* are categorized, based on their aims, into three types: direct, indirect, and supportive measures. Direct measures have a direct impact on the virus and disease prevalence in livestock and humans. Indirect measures have an indirect impact at the prevalence in livestock and humans by reducing transmission and thus prevent further spread of the virus (i.e., to contain the virus). Supportive measures are all measures that are aimed at supporting the implementation of direct and indirect measures so that these measures can achieve their aim(s).

Our framework distinguishes different states, strategies, and measures. It allows a more structured evaluation of measures within a strategy as well as between two or more different strategies based on specific priorities or preferences.

### Step 2: A Systematic Inventory of Measures

Using the developed framework, measures were systematically inventoried to complete the framework for designing the questionnaire and evaluating measures of HPAI control in Indonesia.

Most of the HPAI control programs in livestock and humans in Indonesia were project-based programs funded by other countries or external organizations (e.g., USA, EU, and Australia). The projects are based on national guidelines of HPAI control that were formulated by the government and external organizations (e.g., FAO and OIE). Thus, we collected information for the inventory of measures from the national strategic plan for HPAI H5N1 (5), the USAID report of its HPAI program (18) and Food and Agriculture Organization reports: Avian Influenza control program (19–21) and Emergency Center for Transboundary Animal Diseases (ECTAD) reports (22–25).

In total, we identified and briefly described 35 direct, indirect, or supportive measures for the prevention, monitoring, and control of HPAI since 2004 (see Supplementary Material). Next, these measures were grouped based on their focus, aim, the responsible actors, the targets of measure, the policy level, and year of implementation. Direct and indirect measures were listed under either the prevention, monitoring, or control strategy as defined in the evaluation framework, based on the purpose of each measure.

### Step 3: Questionnaire Development

Based on our framework, we developed a questionnaire aimed at *ex-post* evaluation of the identified HPAI control measures during 2013–2017, and *ex-ante* evaluation of future priorities with regard to direct, indirect, and supportive measures. The questionnaire along with the framework can be used as an additional tool to improve current monitoring and evaluation practice in animal disease management.

The questionnaire was composed in English and translated into Bahasa Indonesia. The first author tested the questionnaire in Bahasa Indonesia with colleagues and then translated the questionnaire back to English for publication.

The questionnaire consists of six parts (Table 1): (i) state of the disease; (ii) priority HPAI impacts; (iii) priority strategic aims; (iv) preferences toward direct, indirect, and supportive measures; (v) the degree of success; and (vi) budget priorities. A sample questionnaire can be obtained from the Supplementary Material. The first five parts of the questionnaire were aimed at *ex-post* evaluation, while the sixth part was aimed at *ex-ante* evaluation. Each part contained a description informing participants of the specific aim.

**Table 1:** Outlines of the questionnaire for the *ex-post* evaluation.

**Framework**	**Purpose of the question**	**Measurement**
1. State of disease	Determine the state of HPAI for each year	A: endemic (high) B: endemic (low)
2. Livestock/ Humans (focus)	Determine priority disease impacts	Ranking
3. Strategy	Determine priority strategic aims	Ranking
4. Measures (ex-post)	Determine priority direct, indirect, and supportive measures (in the past)	Ranking (only for implemented measures)
5. Performance	Evaluate the degree of success, identify key success and not essential measures	Rating (1–3)
6. Budget priority	Determine measures from direct, indirect, or supportive measures to have the highest and lowest budget allocation	Selection of measures

The first part aimed to determine HPAI prevalence in the participant's district (high or low) during the years 2013–2017. As such, we aimed to increase the awareness of the participants in answering subsequent parts of the questionnaire, particularly in determining the priority of measures. In reality, HPAI prevalence in a given district was determined based on the reported cases in the respective administrative area and the definitions highlighted above.

The second and the third part of the questionnaire aimed to determine the rankings of importance for different HPAI impacts and strategic aims for HPAI control. In each part, participants were asked to rank HPAI impacts and strategic aims during 2013–2017.

In the fourth part, we probed for preferred direct, indirect, and supportive measures. Participants were asked to identify from three lists which measures were implemented during 2013–2017. Then, participants were asked to rank measures within each category of measures according to priority. In practice, the list of measures could be adapted to the local context during the questionnaire design process.

In the fifth part, we aimed to evaluate HPAI developments within a district based on four parameters: HPAI prevalence, new cases of HPAI, outbreaks in non-dominant poultry sectors (i.e., layer, ducks, native), and human cases of HPAI. Participants were asked to rate each parameter (1 = worsen, 2 = no change, or 3 = improvement) for each parameter. In practice, this part could be replaced with qualitative information instead.

The sixth and final part handled the consistency with which priority measures were selected. Participants were asked to decide which among all direct, indirect, and supportive measures should receive the highest and lowest budget. Information on budget use and planning could be included. This information is extremely valuable for planners and policy-makers.

### Step 4: Workshop and Evaluation

**Figure 3:**
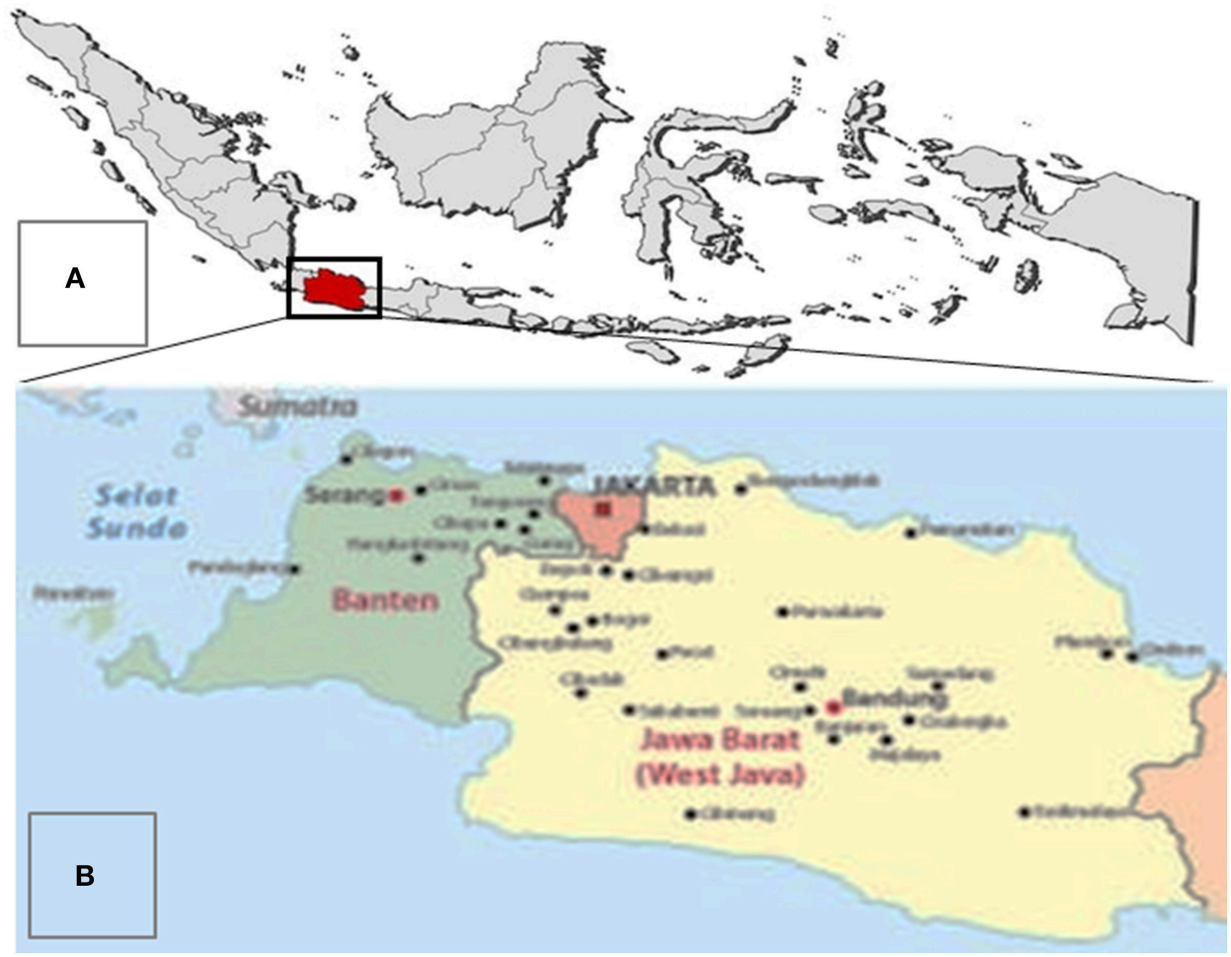
The geographical map of the Republic of Indonesia. **(A)** Provincial boundaries of Indonesia with the West Java Province is highlighted. **(B)** A zoomed map of West Java Province.

The primary purpose of the workshop was to collect expert opinions for the *ex-post* and *ex-ante* evaluation of measures on HPAI control in the West Java province at the district level. In this study, we evaluated HPAI strategies and measures in the context of West Java Province (Figure 3) because West Java has the largest poultry population in the country and accounts for 33% of the national broiler production (26). In addition, the province has been struggling to control HPAI since the first major outbreak of HPAI H5N1 in 2004. Thus, we argue that the results of this study might be useful for other regions in Indonesia that are also in an endemic state. In addition, the workshop was also aimed to present HPAI management framework as a tool to design animal disease control strategies and a framework to evaluate the programs for government officials (i.e., to improve decision-making). The workshop consisted of three activities:

- introduction of the animal health management framework and its use for the evaluation and design of animal disease strategy, particularly in the case of HPAI;
- application of the questionnaire and developed framework to conduct an *ex-post* evaluation of (a) priority disease impacts, strategic aims, and measures, and (b) the degree of success of HPAI control at the district level during 2013–2017; and
- an *ex-ante* evaluation of preferred measures of HPAI mitigation or eradication at the district level.

#### Participating Experts

All participants are active in HPAI management, either as policy-makers or as veterinarians. Moreover, all participants had at least 5 years of professional experience related to HPAI control.

The approach we used for the workshop is the Nominal Group Technique (NGT) (27), which preserves anonymity and ensures an equal contribution of all participants [(28), p. 656]. Instead of implementing the four phases of NGT, the workshop had two phases: individual voting and a round-robin session, because the other two phases, the idea (idea generation) and description of measures (clarification), had been established prior to the workshop [(28), p. 656].

In total, 18 experts (Table 2) were invited to participate in the workshop. We invited local authorities from the top-five districts in terms of poultry population in the West Java province and from the Subang veterinary centers to participate in the workshop. We invited two representatives for each district government (except Subang as a host): the head and senior staff of the animal health department. Representatives from Tasikmalaya were not able to participate, and the empty slots were opened up to additional experts from Subang district. A week before the workshop, an executive summary of the content of the workshop and a short description of the framework were sent to all participants.

**Table 2:** List of experts present at the workshop.

**Regencies/** **Province**	**Department**	**Type**	**Invited**	**Participation**
1. Subang	Animal health	Government	5	7 (two replacement for participants from Tasikmalaya)
2. Tasikmalaya	Animal health	Government	2	–
3. Ciamis	Animal health	Government	2	2
4. Sukabumi	Animal health	Government	2	2
5. Bogor	Animal health	Government	2	2
6. West Java Province	Animal health	Government	2	2
7. Subang	State Polytechnic	Academic	1	1
8. Subang	Veterinary centers	Government	2	2

#### Set-up of the Workshop

A 3 h workshop was conducted on 20 March 2018 in the Veterinary Center of Subang. The workshop was divided into two sessions: a presentation and an evaluation session (with sub-sessions of *ex-post* and *ex-ante* evaluation). The aim of the first session was to present an overview of the study, the objectives, and the framework.

The aim of the second session was to evaluate. For this purpose, participants were divided into two groups consisted of nine participants in each group which is enough and manageable for NGT [(28), p. 656]. Participants from the same institution were separated to avoid discussions between direct colleagues. Participants filled in the questionnaire manually.

The voting session was continued by a round-robin session. In this session, further steps for the planning and implementation of measures were discussed, adjusting currently planned steps. Each participant was encouraged to give his or her comments both verbally as well as in writing.

The second session was followed by a 15-min discussion for additional input and comments.

The workshops were organized in compliance to the codes of ethics for research involving human participants in both Indonesia and the Netherlands. These codes require that participants have to be well-informed about the aims of the research as well as about the anonymity of participants [stated in KNEPK (29); NETHICS (30)]. A short proposal with details of the objectives and the contents of the workshop was sent to all participants before the workshops were held. Before the second session of the workshop, participants were informed about the purposes, and contents of the evaluation session and were asked for their consent. All data were analyzed and reported anonymously. The workshop was conducted by the first and second author together with two facilitators who were trained before the workshop.

### Step 5: Data Analysis

Non-parametric statistics were used to determine the difference between the rankings of measures. We used the Mann-Whitney U test to test the difference of the median scores (i.e., priority or preference) for each pair of HPAI impacts, strategic aims, direct measures, indirect measures, and supportive measures. Measures were ranked based on the difference on the *z*-score. Statistical analyses were performed using SPSS version 23.0 [IBM SPSS for Windows, Armonk, NY: IBM Corp (31)].

## Results

In the workshop, participants were asked to rank HPAI impacts and strategic aims as well as direct, indirect, and supportive measures separately.

Table 3 summarizes the experts' responses and rankings (1 = highest, 7 = lowest) of HPAI impacts during 2013–2017. It is clear that minimizing HPAI impact on public health and production (i.e., increased poultry mortality) were the main concerns of the local authorities. Regarding specific impacts of HPAI, the top priority impacts are human casualties due to HPAI, increased mortality of poultry, and human-HPAI cases. Reducing the morbidity rate for poultry and biosecurity improvement during an outbreak received lower priority. The lowest priority was assigned to loss of market access for farmers and birds due to culling.

**Table 3:** Experts' responses and rankings for HPAI impacts during 2013–2017 (*N* = 17).

**Categories of impacts**	Impacts of HPAI ^*^	**Mean (SE)**	**Mdn**	**Rank**
On-farm (livestock)	Increase in morbidity rate of poultry^b, c, d^	3.82 (0.37)	4	4
	Increase in mortality rate of poultry^a, b^	2.94 (0.35)	3	2
	Improvement of farm biosecurity^d, e^	4.47 (0.36)	4	5
	Loss of birds due to culling^f^	5.94 (0.23)	6	7
Off-farm (livestock)	Loss of market access for the farmers^e, f^	5.41 (0.36)	5	6
Public Health	Human case of HPAI^b, c^	3.06 (0.40)	2	3
	Death case of humans^a^	2.29 (0.57)	1	1

* *means sharing the same superscript are not significantly different from each other (Mann Whitney U, p < 0.05)*.

Table 4 summarizes the experts' responses and rankings (1 = highest, 3 = lowest) for the aims of HPAI control during 2013–2017. The findings suggest that controlling HPAI and protecting the public from HPAI were the top priorities for the local governments. On the other hand, eradicating HPAI was considered a long-term aim, secondary to the top priority aims. In other words, the local governments prioritized public protection from the risk of HPAI transmission by controlling the disease within the poultry chains.

**Table 4:** Experts' responses and rankings for priority strategic aims (*N* = 17).

Aims^*^	**Mean (SE)**	**Mdn**	**Rank**
Mitigation of HPAI^a^	1.65 (0.15)	2	1
Human protection^a^	1.65 (0.21)	1	= 1
Eradication of HPAI	2.59 (0.12)	3	2

* *means sharing the same superscript are not significantly different from each other (Mann Whitney U, p < 0.05)*.

Table 5 summarizes the experts' responses and preference rankings (1 = highest, 5 = lowest) for direct HPAI prevention and control measures. Preventive AI vaccination (*Mean* = 1.35) was ranked significantly higher than the other direct measures, thus, it was considered the top priority direct measure. Direct measures with lower priority are ring vaccination and cleaning and disinfection. Stamping-out and selective depopulation were ranked at lowest priority.

**Table 5:** Experts' responses and rankings for direct, indirect, and supportive measures.

Measures^*^	***N***	**Mean (SE)**	**Mdn**	**Rank**
**DIRECT MEASURES**				
Preventive AI vaccination	17	1.35 (0.21)	1	1
Emergency (ring) AI vaccination^a^	15	2.47 (0.27)	2	2
Poultry restocking (Cleaning & Disinfection)^a, b^	16	2.75 (0.21)	3	3
Stamping-out^a, b, c^	6	3.50 (0.50)	4	4
Selective depopulation^c^	12	3.58 (0.29)	4	= 4
**INDIRECT MEASURES**				
Biosecurity	17	1.24 (0.18)	1	1
Surveillance (villages and farms)^a^	16	2.88 (0.29)	2	2
Zoning and compartmentalization^a, b^	16	3.63 (0.40)	4	3
Traffic control^a, b, c^	8	3.63 (0.71)	4	= 3
Sanitation for transporting vehicles & markets^b, c, d, e^	12	4.50 (0.42)	5	4
Surveillance (wild birds & grazing ducks)^b, c, d, e^	10	4.60 (0.37)	5	= 4
Live bird market (LBM) surveillance^b, c, d^	16	4.75 (0.48)	6	5
Monitoring types of AI virus	8	7.00 (0.42)	8	6
**SUPPORTIVE MEASURES**				
Provision of AI vaccines for sector 3 and 4 farms	16	2.63 (0.64)	1	1
Training farmers about prevention, monitoring, and control of HPAI^a^	15	3.80 (0.55)	4	2
Provision of cold storages for AI vaccines^a, b^	13	4.23 (0.75)	4	3
Community-based AI control^a, b, c^	15	4.33 (0.70)	4	= 3
Good Farming Practices (GFP) training for famers' groups^b, c, d^	15	5.47 (0.55)	5	4
Building and improving animal health posts (Puskeswan)^c, d, e^	16	6.13 (0.64)	6	5
Public communication (mass media, flyers)^d, e, f^	15	6.53 (0.77)	7	6
Monitoring and evaluation^d, e, f, g^	16	7.13 (0.95)	7	7
Supporting facilities for field officers^d, e, f, g, h^	10	7.30 (0.72)	8	= 7
Building and improving animal health laboratories^e, f, g, h, i^	9	7.67 (1.04)	8	= 7
Regulations^d, e, f, g, h, i, j, k^	9	8.11 (1.18)	9	8
Laboratories collaborations^f, g, h, i, j^	14	8.29 (0.63)	8	9
Research^k^	8	10.88 (0.83)	11	10
Provision of Personal Protective Equipment (PPE)^**^	1	5.00 (0.00)	5	11

* *means sharing the same superscript in their respective category (i.e., direct, indirect, and supportive) are not significantly different from each other (Mann Whitney U, p < 0.05)*.

** *excluded from statistical analysis*.

Table 5 also summarizes the experts' responses and preference rankings (1 = highest, 8 = lowest) for indirect prevention, monitoring, and control measures. Biosecurity (*Mean* = 1.24) and monitoring the types of AI virus (*Mean* = 7) were ranked significantly higher and lower, respectively, than other indirect measures, indicating that biosecurity is the top priority among different indirect measures, while monitoring of HPAI virus type is the lowest priority. Indirect measures with lower priority were surveillance at farms and villages, zoning and compartmentalization, and traffic control.

Preference rankings for supportive measures in controlling HPAI are summarized in Table 5 as well, with 1 indicating the highest ranking and 14 the lowest. Provision of AI vaccines for sector 3 and 4 farms (*Mean* = 2.63) was ranked significantly higher than the other supportive measures, suggesting that it is the top priority supportive measure. Lower priority supportive measures (ranking 2–4) include provision of supporting facilities for vaccination programs and training farmers with regard to HPAI control.

Table 6 summarizes the ratings (1 = worsen, 2 = no change/same condition, 3 = improvement) on the condition of each parameter of HPAI state development during 2015–2017. In addition, the perceived degree of success of HPAI control measures was evaluated using four parameters. The results show an overall improvement on all parameters. Human-HPAI cases (*Mean* = 3) were perceived to be reduced. This result is in line with the actual number of reported human-HPAI cases during 2015–2017 (4). In addition, experts also perceived that the number of HPAI outbreaks had decreased on broiler farms (*Mean* = 2.76); on duck, layer, and native chickens farms (*Mean* = 2.76); and (3) in AI-free regions (*Mean* = 2.59). However, the scores also indicate room for improvement as there are districts that still have outbreaks. The scoring may be affected by a lack of information about HPAI outbreaks due to underreporting by farmers and limited surveillance by the government.

**Table 6:** Summary of responses for the degree of success of HPAI control (*N* = 17).

**The degree of success of HPAI control strategies**	**Mean (SE)**	**Rank**
HPAI prevalence on broiler sector	2.76 (0.16)	2
New cases of HPAI in AI-free sub-districts	2.59 (0.17)	3
Outbreaks on duck, layer and native chicken farms	2.76 (0.16)	2
Human-HPAI case	3.00 (0.00)	1

In addition to the *ex-post* evaluation, the *ex-ante* evaluation helped to determine the preferred budget allocations toward different measures within two different budget constraint scenarios. Table 7 summarizes the number of votes on the highest and lowest budget allocation among direct, indirect, and supportive measures. This evaluation aims to look at whether preferences for top priority measures are still consistent when phrased in terms of financial resource allocation.

**Table 7:** List of measures with the number of voting for highest and lowest budget allocation.

**Category**	**Measures**	Budget scenario 1^*^: same		Budget scenario 2^**^: lesser	
		**Highest^**a**^ (*N* = 16)**	**Lowest^**a**^ (*N* = 15)**	**Highest (*N* = 14)**	**Lowest (*N* = 12)**
Direct	Preventive AI vaccination	7	–	7	–
Direct	Stamping-out	2	–	2	–
Indirect	Biosecurity	3	–	1	–
Supportive	Public communication	3	2	3	1
Supportive	Provision of AI vaccines	–	–	1	–
Supportive	Building and improving animal health posts	1	–	–	–
Supportive	Community-based AI control	–	1	–	–
Direct	Selective depopulation	–	–	–	2
Supportive	Regulations	–	1	–	1
Supportive	Monitoring and evaluation	–	1	–	1
Supportive	Training of farmers (prevention and control of HPAI)	–	2	–	1
Direct	Poultry restocking (cleaning & disinfection)	–	3	–	1
Indirect	Zoning and Compartmentalization	–	5	–	5

* *budget scenario 1: similar amount/percentage of budget for HPAI in the future*.

** *budget scenario 2: lower budget for HPAI in the future*.

a *highest/lowest: number of voting for which measures are preferred to have the highest/lowest budget allocation*.

In current budget constraints, measures with the highest budget allocation were preventive AI vaccination (7), biosecurity (3), and stamping-out (2), while the lowest budget allocation went to zoning and compartmentalization (5), poultry restocking (3), training of farmers for prevention and control of HPAI (2), and public communication (2).

In a scenario with more stringent budget constraints, measures with the highest budget allocation were preventive AI vaccination (7), public communication (3), and stamping-out (2). The lowest allocation of budget was for zoning and compartmentalization (5), and selective depopulation (2).

The results show that preventive AI vaccination is consistently rated as a top priority measure. Combining the results of both *ex-post* (Table 5) and *ex-ante* (Table 7) evaluation, we created a list of preferential measures of the government including budget allocation priorities (Table 8). This table lists the priorities from all categories of measures: (1) preventive AI vaccination, (2) biosecurity, and (3) stamping-out. The budget allocation consistently underscores the top priority measures from each category of measures, even though provision of AI vaccines received only one vote. This is because the government usually provides AI vaccines during the wet season or if an outbreak occurs. Although stamping-out is not a priority measure, the compensation can take up a substantial portion of the budget.

**Table 8:** List of priority direct, indirect, and supportive measures for mitigation strategy.

**Measures**	**Priority**	**Budget allocation priority**
**DIRECT MEASURES**		
Preventive AI vaccination	1	Very high
Emergency (ring) vaccination	2	N.A.
Poultry restocking	3	Very low
Stamping-out	4	High
**INDIRECT MEASURES**		
Biosecurity	1	High
Surveillance (village and farms)	2	None
Traffic Control	3	None
Zoning and compartmentalization	3	Very Low
**SUPPORTIVE MEASURES**		
Provision of AI vaccines	1	Mid
Training of farmers for HPAI prevention, monitoring, and control of HPAI	2	Very low
Cold-chain for AI vaccine storages	3	N.A.
Community-based AI control program	3	Low

## Discussion

This study carried out a systematic evaluation of measures directed at HPAI mitigation in the West Java Province of Indonesia. The study was carried out in different steps: development of an HPAI management framework for an endemic state, inventorization of measures, design of a questionnaire, and *ex*-*post* and *ex*-*ante* evaluations of measures through a half-day workshop with experts.

The results of this study show that the animal disease management framework is helpful as a systematic structure to distinguish and evaluate strategies and measures. The use of our framework can also be extended to the evaluation of strategies and measures for other zoonotic infectious diseases within a one health approach. The NGT approach proved to be fruitful for the workshop conducted as part of this study. Results from the first round of voting round were not shown to participants and a second round of ranking/voting could not be conducted because of time constraints. Based on the objective of this study, most participants in the workshop were government officials because the officials have knowledge and experience about the implementation of measures on the fields and are the planners and decision-makers of HPAI strategies within their respective districts. Thus, we did not include other expertise, for instance, representatives of farmer groups who are the subjects of the strategies. In addition, this study did not review the governance of control, the role of the national government, of local governments, and of international organizations that even may fund a part of the control. We argue that a further study which focuses on this particular topic would be interesting.

### HPAI Mitigation Priorities for Local Governments

By carrying out the evaluation, this study identified priority aims, impacts, and preferred measures (i.e., direct, indirect, and supportive) for HPAI control in the West Java Province. Two primary aims of the local government directed to HPAI were identified: protecting humans from HPAI and reducing the prevalence of HPAI on poultry farms. Eradication, which is the legally mandatory aim in most Western countries, was not a main aim for the Indonesian local governments. This implies an HPAI strategy that is aligned with the aim of HPAI mitigation. The priority aims are also reflected in the ranking of disease impacts, where effects on public health and farms (e.g., mortality and morbidity rate) stand out.

The preferred *direct measures* were consistent with the impact and aims. The high preference of vaccination is consistent with the priority of prevention of HPAI infection to birds (i.e., mitigation) and transmission to humans (i.e., protection). Moreover, the low preference given to measures of total and selective culling were consistent with the low priority of an HPAI impact on loss of birds due to culling.

For the *indirect measures*, the high preference of biosecurity was consistent with vaccination. In order to increase the success rate of vaccination, improvement and uptake of biosecurity measures are essential [(32), p. 71]. Lower preference was given to surveillance on farms and villages. Surveillance measures are critical to provide all stakeholders with information about the prevalence of AI and other avian diseases as well as to ensure the proper implementation of vaccination. Such information can be used to stimulate farmers to improve animal health management. Although the monitoring of AI virus types was considered as the least preferred among indirect measures, it is important to note that information with regard to the variation in types or clades of AI virus across different districts may be beneficial for the implementation of suitable prophylactic vaccination. Especially in an endemic state, systematic implementation of vaccination against the same type of HPAI virus circulating in poultry is important [(33), p. 10]. Such information will also aid the development of a more effective AI vaccine, and therefore, a more successful vaccination program.

The findings on preferred *supportive measures* are consistent with the preference for vaccination. Provision of AI vaccines and the availability of cold storage may enable farmer access to HPAI vaccines. Furthermore, supportive measures related to farmer training on HPAI control are also essential to increase the low uptake of proper vaccination and biosecurity measures.

Overall, HPAI control measures with the highest preference, such as preventive vaccination, biosecurity, and stamping-out, coincided with the priority aims of the local government. Measures receiving lower preference were emergency vaccination, surveillance at the village and farm level, and provision of vaccines.

Based on these results, the preferred path for a HPAI control strategy which is in line with the strategy preferences would be vaccination-based mitigation to safeguard human and poultry livestock. This is consistent with the findings from a study on risk factors for poultry outbreak in the West Java Province by Yupiana et al. (12). The authors suggest that the most effective way to prevent the spread of HPAI (i.e., to humans and livestock) is by implementing preventive and control measures on poultry farms. A main subsequent issue, therefore, is whether this preferred strategy is feasible to be practically implemented in West Java. However, one might question how feasible vaccination-based HPAI mitigation is in Indonesia, particularly in West Java.

### Feasibility of Vaccination-Based HPAI Mitigation in West Java Province

Vaccination strategies can only be effective if governments are consistent when implementing measures, even if there is a serious budget constraint. The implementation of vaccination also depends on farmer behavior, as farmers need to implement vaccination measures on their farms. They might do so out of self-interest (i.e., higher benefits than costs) or for the benefit of the public and their relatives (i.e., by preventing HPAI transmission to humans).

For a vaccination-based mitigation strategy to be successful, i.e., to protect humans, the coverage and the efficacy of vaccination as well as the uptake of biosecurity measures should be sufficient [(32), p. 71; (33), p. 8; (34), p. 70; (35), p. 10]. In the Indonesian context, a large improvement on both levels and on a long-term basis is needed.

The *coverage* of HPAI vaccination remains low in Indonesia due to the low uptake of vaccination by farmers. Vaccination is less common in broiler farms due to the short production cycle, particularly in sector 3 and 4 farms, which are the main suppliers in the traditional market channel. Vaccination is more common in layer farms because these farms have appropriate equipment and trained staff [(36), p. 12]. In addition, a single AI vaccination is not sufficient to give full protection for broiler chickens before they are slaughtered [(35), p. 10]. Immunity can only be achieved after two vaccinations, making the process either more expensive or less effective. Therefore, some farmers do not favor vaccination. In contrast, poultry farmers working with a longer production cycle, i.e., native and layer chicken farmers, may have a more positive attitude toward vaccination, as the benefit of vaccination can outweigh the implementation cost. The total coverage of HPAI vaccination is far from sufficient.

Regarding the *efficacy* of AI vaccines, several studies have shown that AI vaccines are not effective to prevent the spread of HPAI H5N1 in broiler and layer chickens [(35), p. 10; (37), p. 639; (38), p. 9–12). The efficacy of vaccines also partially depends on how well they target certain strains of AI virus, for instance. Thus, production and provision of AI vaccines that are suitable for various strains of HPAI virus is essential to increase the efficacy of any vaccination program. Governmental monitoring and surveillance activities can help to identify which specific strains of AI virus circulate in a particular region and, consequently, a suitable vaccine can be identified for local use.

Increasing the efficacy of vaccination also requires a *proper implementation* of vaccination. In the context of West Java Province, the broiler production sector includes a four different farm types, that can be categorized, based on their level of biosecurity (FAO). Large industrial integrated broiler farms with high biosecurity (sector 1); medium- to large-scale commercial broiler farms with moderate to high biosecurity (sector 2); small- to medium-scale commercial broiler farms with low biosecurity (sector 3); and backyard broiler farms with low biosecurity and a small number of birds per farm (sector 4) [(36), p. 9]. Although the sector 1–4 categorization started to be used for biosecurity reasons, it is often also used for a position in the value chain. Sector 1 farms always market their chickens in the modern channel; sector 2 farms mostly market their chickens in the modern channel but sometimes to the traditional channel through private collecting farms; and sector 3 and 4 farms always market their chickens in the traditional channel [(39), p. 6]. Farms in sectors 1 and 2 have more capabilities and resources to conduct a proper vaccination as well as to control the disease in case of an outbreak. As a consequence, the implementation of vaccination is often lacking or poorly implemented in sector 3 and 4 farms. This condition is exacerbated by the fact that there is a market for sick poultry, reducing the economic consequences of HPAI outbreaks for broiler farms. This may in turn reduce the motivation of farmers to vaccinate and improve biosecurity, hampering HPAI control in Western Java [(39), p. 10]. Thus, vaccination to control HPAI need more emphasis for sector 3 and 4 farms rather than sector 1 and 2 farms.

In the context of the West Java Province, vaccination-based HPAI mitigation requires a more active collaboration between government, integrated companies, and farmers in different districts. Such collaborations can open opportunities for new HPAI mitigation schemes. Certain schemes may stimulate stakeholders to act in the public interest, i.e., to control HPAI. As a result, achieving the aims to safeguard both humans and birds from HPAI would become more feasible. Although desirable, vaccination-based mitigation strategies were found to be inefficient in Indonesia where there are many sector 3 and 4 farms spread in rural regions. Therefore, an alternative strategy has to be considered.

### Alternatives to Control HPAI

Vaccination-based HPAI mitigation may result in endemicity and antigenic drift of the viral strain [(33), p. 1]. A non-vaccination mitigation strategy could be considered as a second alternative, targeted to the actors in the value chain.

One might consider prioritizing biosecurity measures to mitigate the spread of HPAI [(40), p. 7]. The improvement of biosecurity measures on sector 3 and 4 farms can focus on basic measures such as sanitation of the farm and the personnel, and strict access control to the farm. Biosecurity measures are poorly implemented in sector 3 and 4 farms in Indonesia [(36), p. 9; (39), p. 8]. However, basic biosecurity measures alone do not necessarily reduce the mortality rates in poultry within a backyard setting [(41), p. 650–654]. Thus, vaccination and monitoring and surveillance measures for farms and villages need to be retained in the strategy but will receive lower priority. Monitoring the implementation of biosecurity measures is essential for the correct application of measures as well as to shape new habits with farmers.

Biosecurity measures need to be applied not only by farmers, but also other stakeholders such as buyers, for instance by disinfecting the cars and the crates that are used to transport poultry. Modifying transportation cars into semi-closed vehicles with a fan may also help to reduce the spread of HPAI during transport of live birds.

## Conclusions

From this research, we conclude that the aim of the local governments in West Java is to protect humans and livestock from HPAI. Governmental experts prefer vaccination-based mitigation to safeguard humans and poultry. Eradication is considered a long-term goal.

Based on the aim, selected strategies are identified: mitigation and human protection. Coinciding with the priority aims, the top preferred measures identified by the experts are: vaccination, biosecurity, stamping-out, and provision of AI vaccines. However, the feasibility of vaccination-based HPAI mitigation is low, particularly in sector 3 and 4 broiler farms (e.g., low efficacy of vaccines and limited uptake of measures). A collaboration between the government, integrated companies, and farmers may help to increase the feasibility of either a vaccination- or biosecurity-based mitigation strategy.

## Author Contributions

MP designed the study, collected, and analyzed the data, and drafted the manuscript. The remaining authors provided input on the design of the study, helped in interpreting study results, and critically revised the manuscript. All authors read and approved the final manuscript.

### Conflict of Interest Statement

The authors declare that the research was conducted in the absence of any commercial or financial relationships that could be construed as a potential conflict of interest.
