# Supplementary material for: A Systematic Evaluation of Measures Against Highly Pathogenic Avian Influenza (HPAI) in Indonesia
Source: Front Vet Sci. 2019 Feb 18;6:33. doi: 10.3389/fvets.2019.00033 (PMC6387902; doi:10.3389/fvets.2019.00033)
Supplement: Supplementary file 2 [file Table_2.DOCX]

## PART 1: Ex-post evaluation

1. **The state of HPAI (H5N1) disease in your regency during the last 5 years**
2. We have three categories, namely proportion of infected sub-districts, frequency of outbreaks, and incidences/new outbreaks to determine whether your regency is either in a ***high*** or ***low*** prevalence of HPAI :

| **Category** | **Endemic (high)** | **Endemic (Low)** |
| --- | --- | --- |
| Proportion of infected sub-districts (prevalence) | Annually, reports/issues of a massive and sudden death of birds within **more than 15 sub-districts *or* > 50% of sub-districts** of the regency. | Annually, reports/issues of a massive and sudden death of birds within **less than 15 sub-districts *or* < 50% of sub-districts** of the regency. |
| Frequency of outbreaks | **Similar and/or higher frequency** of reports/issues of a massive and sudden death of birds and/or outbreaks compared to **the past 1-2 years.** | **lower frequency of** reports/issues of a massive and sudden death of birds and/or outbreaks compare to **the past 1-2 years.** |
| Incidences/New outbreaks | Incidences/new outbreaks **still occur** in sub-districts which did not have any outbreak in the past 1-2 years. | **no more incidence** occurred in sub-districts which did not have an outbreak in the past 1-2 years. |

Question: According to your knowledge/experience and/or information you have, what is the status **(endemic: high *or* low)** of the HPAI in your regency during the last 5 years?

*(write* ***A*** *for endemic (high)* ***B*** *for endemic (Low) for each year)*

| Year | 2013 | 2014 | 2015 | 2016 | 2017 |
| --- | --- | --- | --- | --- | --- |
| **A (high)/B (low)** |  |  |  |  |  |

1. Question: are there sub-districts/areas in your regency that have a compartment? *(tick (****V****) the box)*

- Yes, How many: ______compartments Type of farm: _______
- No

1. **Most important impacts of HPAI (H5N1) in your regency during the last 5 years**
2. Question: We would like to know, according to your opinion, the relative importance of various impacts of HPAI as written on the table below. *You can rank the importance of each impact from 1 to 7 (1= most important, 7=least important).*

| **No.** | **Category** | **Sub-category** | **Impacts** | **Ranking** |
| --- | --- | --- | --- | --- |
| 1 | Livestock (on farm) | Resources (birds) | Increasing morbidity rate of (sick) poultry birds |  |
| 2 |  |  | Increasing mortality rate of poultry birds on farms |  |
| 3 |  | Control Measures | More farms improve their biosecurity measures |  |
| 4 |  |  | Loss of birds due to selective depopulation/culling |  |
| 5 | Livestock (off-farm) | Market | Loss of market access for the farmers |  |
| 6 | Humans/Public Health |  | The case of humans who got ill due to HPAI H5N1 |  |
| 7 |  |  | Death cases of human due to HPAI H5N1 |  |

1. **Strategic Aims on HPAI (H5N1) management in your regency during the last 5 years**
2. Question: We would like to know how you rank the importance of these three aims in your regency. *Please rank these strategic aims based on the importance of each aim (give a ranking in each cell by writing 1, 2, and 3; 1= most important, 2= important, 3= least important)*

| No. | **Aims** | **Definition** | **Ranking** |
| --- | --- | --- | --- |
| 1 | **Mitigation of HPAI** | Reducing the virus prevalence or the number of AI outbreaks to the extent that control measures can be effectively done to eradicate the virus |  |
| 2 | **Eradication of HPAI** | Eradicating the disease/virus to achieve AI-free status |  |
| 3 | **Human protection** | Reducing the risk of transmission of HPAI H5N1 virus to human and treating suspected human patients. |  |

1. **Most important measures that have been taken in your regency for HPAI during the last 5 years**
2. Question: We would like to know which **direct measures** that have been implemented in your regency during the last 5 years and the importance of the measures.

*Circle* ***(O)*** *either* ***Yes*** *or* ***No*** *to determine your answer.*

*Give ranking only to the measures that you have implemented in the last 5 years with 1 as the most important measure, if you choose* ***(NO)*** *for a particular measure, then give* ***(-)*** *for the ranking.*

| **No.** | **Direct Measure** | **Category** | **Definition** | **Yes/No** | **Ranking** |
| --- | --- | --- | --- | --- | --- |
| 1 | Preventive AI vaccination | Prevention | Vaccinate birds to prevent AI outbreak | Yes / No |  |
| 2 | Emergency (ring) AI vaccination | Control | Give emergency vaccination to all sector 4 farms within 3 KM radius of the site of AI outbreak | Yes / No |  |
| 3 | Culling/Stamping-out | Control | Kill all healthy and infected birds in the infected farm(s) | Yes / No |  |
| 4 | Selective depopulation | Control | Only kill the suspected and infected birds on the infected farm | Yes / No |  |
| 5 | Poultry restocking (Cleaning & Disinfection) | Control | cleaning and disinfecting infected farm, leave it empty for 3 weeks | Yes / No |  |

1. Question: We would like to know which **indirect measures** that have been implemented in your regency during the last 5 years and the importance of the measures.

*Circle* ***(O)*** *either* ***Yes*** *or* ***No*** *to determine your answer.*

*Give ranking only to the measures that you have implemented in the last 5 years with 1 as the most important measure, if you choose* ***(NO)*** *for a particular measure, then give* ***(-)*** *for the ranking.*

| **No.** | **Indirect Measure** | **Category** | **Definition** | **Yes/No** | **Ranking** |
| --- | --- | --- | --- | --- | --- |
| 1 | Biosecurity | Prevention | Disinfecting sector 3 and/or 4 farms | Yes / No |  |
| 2 | Surveillance (village and farms) | Monitoring | Surveillance of AI virus on sector 3 and 4 poultry farms | Yes / No |  |
| 3 | Surveillance (Wildlife and grazing ducks) | Monitoring | Monitor the movement of ducks and bird migration | Yes / No |  |
| 4 | Live bird market surveillance | Monitoring | Surveillance of AI virus on live bird markets/traditional markets | Yes / No |  |
| 5 | Monitoring the clade/type of AI virus | Monitoring | Surveillance of the development of AI virus strains or clade | Yes / No |  |
| 6 | Sanitation within the poultry chain (vehicles and market) | Control | Providing facilities for trucks/vehicles to clean and Cleaning + Disinfecting traditional/live bird markets | Yes / No |  |
| 7 | Traffic control of live birds and poultry products | Control | Controlling the vehicles (truck) carrying live birds and poultry products on the district borders | Yes / No |  |
| 8 | Zoning and compartmentalization | Control |  | Yes / No |  |

1. Question: We would like to know which **supportive measures** that have been implemented in your regency during the last 5 years and the importance of the measures.

*Circle* ***(O)*** *either* ***Yes*** *or* ***No*** *to determine your answer.*

*Give ranking only to the measures that you have implemented in the last 5 years with 1 as the most important measure, if you choose* ***(NO)*** *for a particular measure, then give* ***(-)*** *for the ranking.*

| **No.** | **Category of Supportive measures** | **Supportive measures** | **Yes/No** | **Ranking** |
| --- | --- | --- | --- | --- |
| 1 | HPAI control supports | Provision of AI vaccines to small-scale and backyard farmers | Yes / No |  |
| 2 |  | Provision of Personal Protective Equipment (PPE) for farmers | Yes / No |  |
| 3 |  | Providing cold chain for AI vaccines storage | Yes / No |  |
| 4 | Capacity building | Building and improving animal health posts (Puskeswan) | Yes / No |  |
| 5 |  | Building and improving animal health laboratories | Yes / No |  |
| 6 |  | Supporting facilities (vehicles, phones, GPS) for field officers | Yes / No |  |
| 7 | Public Private Partnership (PPP) | Training of Good Farming Practices to farmers groups | Yes / No |  |
| 8 |  | Training farmers on how to conduct proper prevention, monitoring and control of HPAI | Yes / No |  |
| 9 |  | Laboratories collaboration | Yes / No |  |
| 10 | Risk communication, information, and awareness | Community-based AI control program | Yes / No |  |
| 11 |  | Public communication (posters, radio, newspaper) | Yes / No |  |
| 12 | Research and Development | Research related to HPAI | Yes / No |  |
| 13 | Regulations | Regulations related to HPAI prevention and control | Yes / No |  |
| 14 | Monitoring and evaluation | Monitoring and evaluation of progress on HPAI | Yes / No |  |

1. **The degree of Successfulness and contribution of measures to the regency achievement**
2. Question: Referring to the development of HPAI in your regency during the last 3 years, how is the change of HPAI case in your regency.

*Circle (****O****) the answer (1, 2, or 3)*

| **No.** | **Category** | **Definition** | **Reduction** | **Relatively no change** | **Improvement** |
| --- | --- | --- | --- | --- | --- |
| 1 | HPAI prevalence | Massive and sudden death of birds on **broiler farms** | more outbreaks or news or reports on farms | The number of incidence or outbreaks or reports relatively stays the same | lesser outbreaks or news or reports on massive sudden death of birds on a farm |
|  | Answer (circle) | | 1 | 2 | 3 |
| 2 | New cases of HPAI | In overall, **incidences/new outbreak in sub-districts** that did not have AI outbreaks in the past 1-2 years | The (total) number of newly infected sub-districts increases | The (total) number of newly infected sub-districts stays the same as the previous year | No newly infected sub-districts or fewer number of newly infected sub-districts |
|  | Answer (circle) | | 1 | 2 | 3 |
| 3 | Outbreaks in other sector of poultry farms | Massive and sudden death of birds on **other poultry farms (e.g. layer, ducks, native)** | More/new outbreaks or reports on other species of poultry birds (e.g. ducks, layer, native farm) | The number of incidence or outbreaks or reports on other species of poultry birds relatively stays the same | no/lesser outbreaks on other species of poultry birds (e.g. ducks, layer, native farm) |
|  | Answer (circle) | | 1 | 2 | 3 |
| 4 | Human-AI case | Cases where a human is infected by HPAI | More human-AI cases | The number of human cases stays the same | No more/lesser human-AI case |
|  | Answer (circle) | | 1 | 2 | 3 |

1. Description: for the evaluation to be more useful for your agency/regency, it is also important to identify direct, indirect, and supportive measures that:

- **essentials for the success**, or ,in other words, a measure(s) that if you remove from your strategy will suffer the achievement a lot.
- **have no impact to the success and achievement of your strategy** even if you remove these measures from your strategy; and/or measures that **should be changed/improved** to give impact to the success of your strategy.

Question: Referring to the list of measures **(No.4)** that you have identified, please fill the table below on which direct, indirect and supportive measures fit to the category written below.

***Write only the number (left side of the table on No. 4.1, 4.2, and 4.3) of the measures****. You can fill a cell with more than one measure, but cannot put the same measure in another row. If you think that there is no measure to be written in a cell you can write* ***(-)****.*

| **No.** | **Category** | **Direct measures** | **Indirect measures** | **Supportive measures** |
| --- | --- | --- | --- | --- |
| 1 | Measures that is essential for the success |  |  |  |
| 2 | Measures that have no impact; can be skipped and/or should be changed/improved |  |  |  |

## PART 2: Ex-ante evaluation

1. Question: for improving mitigation or eradication of HPAI in your regency, what are measures that would be kept, dropped, and added assuming the budget allocation for animal health, particularly for AI, **remains the same**? (Note: If you add a new measure, then you have to drop another measure)

In case you have measures that you kept and added, which measure will get the most and lowest budget? *(****one measure for the measure with most as well as least budget****)*

*Please fill in each cell with the measures that you have identified in* ***No.4* only with the number of the measure***.*

| **No.** | **Scenario (same)** | **Direct measures** | **Indirect measures** | **Supportive measures** |
| --- | --- | --- | --- | --- |
| 1 | Measures that will have most budget ( write the number of the measure from the list of measures that you kept and added) |  |  |  |
| 2 | Measures that will have lowest budget ( write the number of the measure from the list of measures that you kept and added) |  |  |  |

1. Question: for improving mitigation or eradication of HPAI in your regency, what are measures that would be kept, dropped, and added assuming the budget allocation for animal health, particularly for AI, is **cut/reduced**? (Note: If you add a new measure, then you have to drop another measure)

In case you have measures that you kept and added, which measure will get the most and lowest budget? *(****one measure for the measure with most as well as least budget****)*

*Please fill in each cell with the measures that you have identified in* ***No.4* only with the number of the measure***.*

| **No.** | **Scenario (same)** | **Direct measures** | **Indirect measures** | **Supportive measures** |
| --- | --- | --- | --- | --- |
| 1 | Measures that will have most budget ( write the number of the measure from the list of measures that you kept and added) |  |  |  |
| 2 | Measures that will have lowest budget ( write the number of the measure from the list of measures that you kept and added) |  |  |  |
